# Effects of lighting schedule during incubation of broiler chicken embryos on leg bone development at hatch and related physiological characteristics

**DOI:** 10.1371/journal.pone.0221083

**Published:** 2019-08-15

**Authors:** Carla W. van der Pol, Inge A. M. van Roovert-Reijrink, Sander W. S. Gussekloo, Sander Kranenbarg, Karen M. Leon-Kloosterziel, Margaretha H. van Eijk-Priester, Michal Zeman, Bas Kemp, Henry van den Brand

**Affiliations:** 1 Research department, HatchTech B.V., Veenendaal, the Netherlands; 2 Adaptation Physiology Group, Wageningen University and Research, Wageningen, the Netherlands; 3 Experimental Zoology Group, Wageningen University and Research, Wageningen, the Netherlands; 4 Department of Animal Physiology and Ethology, Faculty of Natural Sciences, Comenius University Bratislava, Bratislava, Slovak Republic; 5 Institute of Animal Biochemistry and Genetics, Slovak Academy of Sciences, Bratislava, Slovak Republic; Pennsylvania State University, UNITED STATES

## Abstract

Providing a broiler chicken embryo with a lighting schedule during incubation may stimulate leg bone development. Bone development may be stimulated through melatonin, a hormone released in darkness that stimulates bone development, or increased activity in embryos exposed to a light-dark rhythm. Aim was to investigate lighting conditions during incubation and leg bone development in broiler embryos, and to reveal the involved mechanisms. Embryos were incubated under continuous cool white 500 lux LED light (24L), continuous darkness (24D), or 16h of light, followed by 8h of darkness (16L:8D) from the start of incubation until hatching. Embryonic bone development largely takes place through cartilage formation (of which collagen is an important component) and ossification. Expression of genes involved in cartilage formation (*col1α2*, *col2α1*, and *col10α1*) and ossification (*spp1*, *sparc*, *bglap*, and *alpl*) in the tibia on embryonic day (ED)13, ED17, and at hatching were measured through qPCR. Femur and tibia dimensions were determined at hatch. Plasma growth hormone and corticosterone and pineal melatonin concentrations were determined every 4h between ED18.75 and ED19.5. Embryonic heart rate was measured twice daily from ED12 till ED19 as a reflection of activity. No difference between lighting treatments on gene expression was found. 24D resulted in higher femur length and higher femur and tibia weight, width, and depth at hatch than 16L:8D. 24D furthermore resulted in higher femur length and width and tibia depth than 24L. Embryonic heart rate was higher for 24D and 16L:8D in both its light and dark period than for 24L, suggesting that 24L embryos may have been less active. Melatonin and growth hormone showed different release patterns between treatments, but the biological significance was hard to interpret. To conclude, 24D resulted in larger leg bones at hatch than light during incubation, but the underlying pathways were not clear from present data.

## Introduction

There are indications that providing a broiler embryo with a lighting schedule during incubation has a positive effect on embryonic leg bone development and leg health in later life. Huth and Archer [1] incubated White Leghorns under white LED light with a schedule of 12 hours of light, followed by 12 hours of darkness (12L:12D). As a control, they used continuous darkness (24D) throughout incubation. They found fewer chicks with leg abnormalities and that were too weak to stand at hatching for chicks incubated under 12L:12D compared to 24D. However, Archer et al. [2] did not see an effect of 12L:12D of a combination of white and monochromatic red LED light on leg defects in layer and broiler hatchlings when compared to 24D. For embryos incubated under 12L:12D of white LED light we previously found a higher rate of ossification of the tibia (tibiotarsus) between embryonic day (ED)12 and ED14, higher cortical area, cortical thickness, and second moment of area of the tibia, and a longer tibia and femur at hatch compared to continuous light (24L), with 24D mostly intermediate [3].

Bones with higher cortical thickness and a higher second moment of area are probably stronger [4], and more resistant to stress fractures and bending than bones with lower cortical thickness [5]. This is relevant for broiler production, because the body conformation of broilers results in high body weight load on the legs. When body weight load is disproportionate to bone mass and strength, this may result in bones that are relatively not strong enough, and make it more susceptible to leg pathologies, which are a major welfare problem in broiler chickens (reviewed by Bradshaw *et al*. [6]). Stimulating embryonic leg bone development can therefore contribute to improved leg health in broiler chickens.

As we found increased ossification in 12L:12D in our previous research compared to 24L [3], we were interested to see whether genes involved in cartilage and bone development would be up- or down-regulated by light during incubation. First skeletal formation in the avian embryo takes place through the formation of a cartilage model, which becomes calcified from ED9 onward [7–9]. Collagen is the main component of cartilage, and several genes are associated with its formation, of which three are investigated in more detail. In chickens, collagen type I limits expansion of the diameter of the cartilage core [7,10]. The cartilage matrix becomes calcified, and the cartilage starts to change to a bone-forming surface. In chickens, type II collagen is primarily found in chondrocytes in the proliferative zone and in the articular zone of long bones [11]. Chicken collagen type X is synthesized by hypertrophic chondrocytes during endochondral ossification [12]. This type of collagen facilitates endochondral ossification by regulation of the matrix mineralization, and it was found that collagen type X is a reliable marker for new bone formation [13].

Ossification of the cartilage model takes place through the involvement of several bone cell related proteins. In chickens, osteopontin and osteocalcin play a role in extracellular matrix formation [14]. Osteopontin was found in the columns of chondrocytes at the bone’s epiphyseal plate [11], where it marked the onset of differentiation and calcification of the chondrocytes. Osteocalcin is considered to be a phenotypic marker of osteoblasts, which are the bone building cells [15], and it has high binding affinity with calcium [16]. Osteocalcin seems to appear during new bone formation when new osteoblasts begin to proliferate [15]. Osteonectin is produced by chondrocytes [17] and osteoblasts [18], and it accumulates in the mineralizing zone in bone formation [17]. Alkaline phosphatase is another phenotypic marker of bone formation [15]. It is found in the hypertrophic zone of the epiphyseal plate [19], where it is produced exclusively by mature, differentiated chondrocytes [11]. A reduction in alkaline phosphatase could therefore be a sign of delayed chondrocyte differentiation [11], which could indicate a delay in the development of the bone.

It can be speculated that embryonic bone development is affected by light during incubation through the involvement of melatonin, a hormone that is released from the pineal gland during dark periods [20]. For example, scoliosis was found to develop in all 30 chickens that were pinealectomized 3 days after hatching by Machida *et al*. [21]; but when these chickens received melatonin injections every other day, only 6 out of 30 chickens developed scoliosis. However, in a previous study, we did not find differences in plasma melatonin between lighting schedules in the period between ED18.8 and ED19.5 [3]. Other authors did see a clear dark-light dependent rhythm of plasma melatonin from ED18 onward for chickens incubated under 16L:8D [22] and 12L:12D [23], but did not study its direct effects on bone development. Possibly, a circadian rhythm in melatonin levels can be discovered when the pineal gland, where the majority of melatonin is produced, is sampled directly. There are indications that growth hormone (GH) release is stimulated by melatonin, as GH was found to peak along with melatonin in male rats [24]. Also, green LED light during incubation has been demonstrated to increase GH levels in the plasma of ED15 embryos [25], in newly hatched chicks [26,27], and up till day 5 post hatching [27]. In chickens, it appears that GH stimulates chondrocyte proliferation [28]. Corticosterone acts as a negative regulator of bone development through increasing collagen degradation [29] and decreasing chondrocyte [30] and osteoblast proliferation [31]. Possibly, GH shows a similar circadian release pattern to what we expect in melatonin, while corticosterone could reflect a possible degree of stress from being under- or overexposed to light.

Activity is also involved in leg bone development in embryos. Post hatch, broiler activity is accepted to be of major importance in leg bone development [6]. Embryonic activity may already be driving development of the broiler during incubation. The development of several bones [32] and joints [33] has been shown to be retarded in chicken embryos that were experimentally paralyzed by botulin toxin. Chicken embryos normally exhibit the first movement through muscle contractions by ED4 [34]. It has been suggested that heart rate reflects embryonic movement [35].

Aim of this study was to investigate effects of lighting conditions on leg bone development in broiler chicken embryos, and to reveal the mechanisms involved in differences in bone development. We used a schedule of 16L:8D, which is similar to the post hatch broiler house lighting conditions, and which we hoped would therefore mimic a natural lighting schedule that broilers would experience in their post hatching life. Additionally, [22] found that 16L:8D created plasma melatonin rhythmicity in chicken embryos. 24D was chosen as the current industry’s standard for incubation and 24L was used as the most extreme contrast of 24D.

We hypothesized that 16L:8D would result in upregulation of genes involved in cartilage and bone development, a clear circadian rhythm in pineal melatonin and plasma GH release, lower heart rate as a result of increased embryonic activity, and increased bone development at hatch compared to 24L in particular with 24D intermediate, as the strongest contrast in embryonic bone development was previously found between 24L and a circadian rhythm in light during incubation.

We did find an effect of lighting conditions, but the involved mechanisms and pathways have not yet completely been revealed by the current experiment.

## Materials and methods

The Institutional Animal Care and Use Committee of Wageningen University & Research (Wageningen, the Netherlands) approved the experimental design and protocols.

### Experimental setup

**Fig 1:**
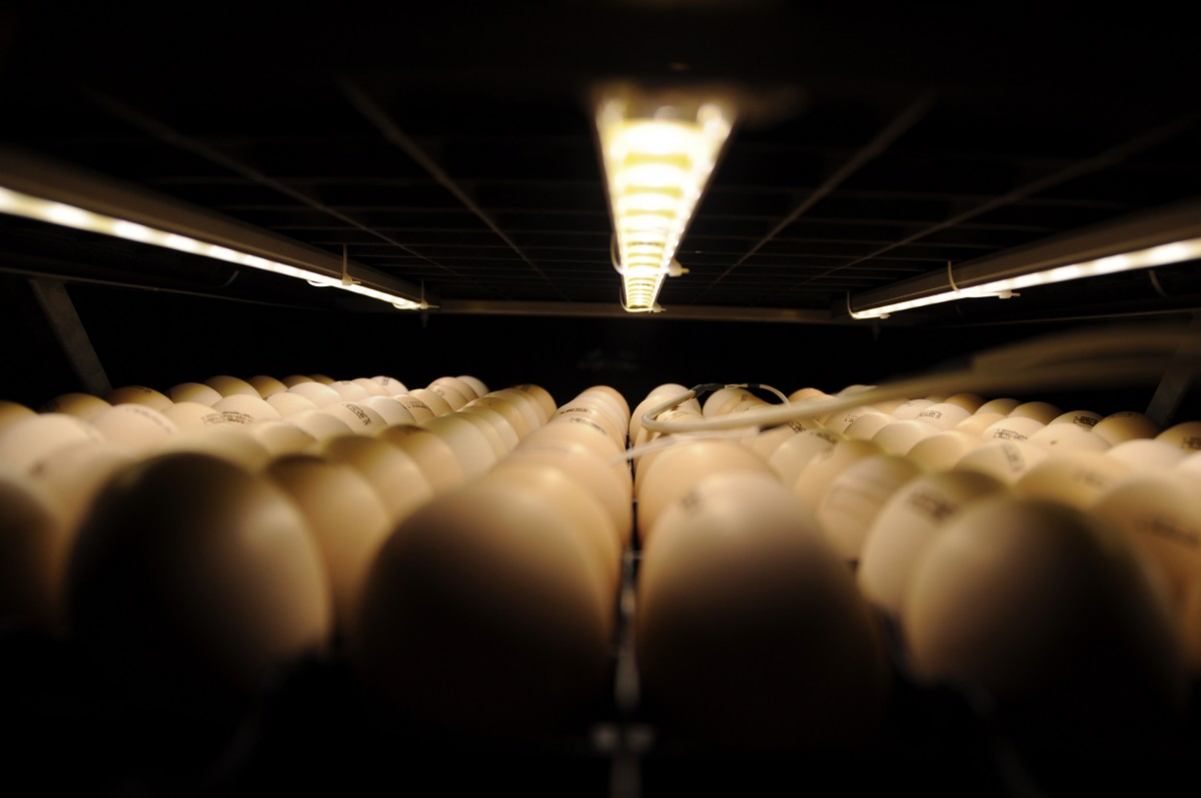
Example of the setup with three LED strips over one tray of eggs in the lighted treatments.

Ross 308 broiler eggs from a 42-week-old parent flock were selected for egg weight between 62.0 and 65.0 g (to reduce variation among eggs). Assuming a hatchability of 80%, 987 eggs were selected to obtain the embryos and chickens required in the experiment and those required for the experiment described in van der Pol *et al*. [36], which ran simultaneously. They were then stored at a commercial hatchery (Lagerwey BV, Lunteren, the Netherlands) for three days at 18°C before being transported to the climate respiration chambers (CRCs [37]) at the experimental facility of Wageningen University & Research (Wageningen, the Netherlands). At the experimental facilities, eggs were allocated to one of three CRCs, each containing 329 eggs and programmed for one of the following treatments: incubation under continuous light (24L), incubation under 16 h of light, followed by 8 h of darkness (16L:8D), or incubation under continuous darkness (24D). Light was provided by cool white light emitting diode (LED) strips with a colour temperature of 6,050 K in a wavelength range of 420 to 780 (peak at 454 nm). According to Yu et al. [38], this range has 7–45% transmittance in the egg. The intensity of the light was approximately 500 lux at eggshell level. 3 LED strips were attached to each egg tray above the experimental eggs (Fig 1). A head torch with low light intensity of approximately 10 lux at eggshell level was used in dark conditions for checks and during measurements. No other light was allowed to enter the CRCs. 16L:8D’s lighting treatment started with 12 h of light, followed by the first dark period of 8 h.

CRCs are built for respiration research, and they are therefore fully air tight (>99.5%) and designed and maintained to allow for very close climate control. At the start of incubation, eggs were randomly divided over 3 setter trays per treatment, and turned to an initial angle of 45°. Eggs were then turned by 90° hourly. Eggs were heated from storage temperature to incubation temperature over a time frame of 10 h. From then on, eggs were incubated at a set eggshell temperature of 37.8°C till ED19.5, measured through 5 temperature sensors (NTC Thermistors: type DC 95; 142 Thermometrics, Somerset, UK) attached to the equator of 5 random eggs per treatment using heat conducting paste (Dow Corning 340 Heat Sink Compound, Dow 144 Corning GmbH, Wiesbaden, Germany) and a small piece of tape. An eggshell temperature of 37.8°C was found to result in the most optimal balance between bone development, hatchability, and chick quality [39]. The median of the 5 temperature measurements from the sensors were used to automatically adjust the CRCs air temperature. Air temperature itself was measured by five temperature sensors per CRC (1/3 DIN-B PT100; accuracy of ≤±0.1°C). CO_2_ levels were measured using two Itron Delta 2050 MP series, type G100 gas flow meters (accuracy of ≤±0.5%), and levels were not allowed to rise above 0.35%. RH was maintained between 45 and 55% throughout incubation as measured through Novasina Hygrodat 100 –HIA 13/20M combi sensors.

On ED19.5, eggs were transferred to hatching baskets by hand. Eggs were not candled for embryonic mortality or fertility to exclude the influence of light on 24D and 16L:8D’s dark period as much as possible. At ED19.5, the machine’s air temperature was fixed at its current setting, since hatching could start from that point onward, and eggshell temperatures were no longer reliable once the chicken has emerged from the egg. At 510 h of incubation or ED21.25, the experiment was ended.

### Embryonic heart rate

Heart rate was measured for the first time on ED12, and for the last time on ED19 at 20:00. It was measured using a Buddy Mk2 digital egg monitor (Avitronics, Truro, United Kingdom) in 10 randomly chosen per treatment eggs twice a day: at 8:00, during 16L:8D’s dark period, and at 20:00, during 16L:8D’s light period. The Buddy Mk2 digital egg monitor uses infrared light and disturbances in its transmission to measure blood pulses [40].

### Quantitative real-time PCR (qRT-PCR)

On ED13, ED17, and at hatch, six randomly selected eggs or chickens per treatment (N = 54) were sampled for tibial gene expression relevant to bone development. Eggs and chickens were kept in their treatment lighting conditions until sampling. Embryos or chickens were sacrificed by cervical dislocation, and the left leg was removed at the femoral head. The tibia was chosen for gene expression measurements, because a previous study showed differences in ossification in the tibia between incubation lighting treatments from ED13 onwards [3]. The tibia was separated from the leg, the fibula was removed, and soft tissue was removed using dry paper. Immediately after dissection, tibias were placed in tubes, snap frozen in liquid nitrogen, and stored at -80°C.

RNA was obtained from the complete tibia using the RNeasy fibrous tissue mini kit (Qiagen, Germany). The bones were homogenized in RLT buffer using a TissueLyser II (Qiagen, Germany). RNA concentration was measured with a NanoDrop 1000 (ThermoFisher Scientific, USA). From each treatment, a few samples were randomly chosen to evaluate the integrity of extracted RNA in gel electrophoresis. cDNA was synthesized from 1 μg total RNA using the Quantitect Reverse Transcription Kit (Qiagen). Gene-specific primers were designed using primerBLAST (www.ncbi.nlm.nih.gov) (Table 1).

**Table 1:** Proteins and their encoding genes, sequences, codes, and primers.

Protein	Gene symbol	Sequence	Primer sequence
Collagen type I alpha 2 chain	*col1α2*	NM_001079714.2	F: AAACCAGGCGAAAGGGGTCT
			R: GATGGACCACGGCTTCCAAT
Collagen type II alpha 1 chain	*col2α1*	NM_204426.1	F: GACCGCGACCTCCGACAA
			R: CCTCGGGGTCCTACAACATC
Collagen type X alpha 1 chain	*col10α1*	XM_003641007.3	F: CCACAACATTTGAGGACGGA
			R: CCCCTTGATGCTGGACTGTT
Osteopontin (secreted phosphoprotein 1)	*spp1*	NM_204535	GAAAAATACGACCCCAGGAGC
			TGCTGAAGTGAAGCCAGGTC
Osteonectin (secreted protein acidic and cysteine rich)	*sparc*	NM_204410.1	ACTGCACCACTCGCTTCTTT
			ATGTCCTGCTCCTTAATGCCAA
Osteocalcin (bone gamma-carboxyglutamate protein)	*bglap*	NM_205387.1	TAAAGCCTTCATCTCCCACCG
			TCAGCTCACACACCTCTCGT
Alkaline phosphatase	*alpl*	NM_205360.1	GTCAAAGCCAACGAGGGGAC
			TTCATCCTTAGCCCAGCGGA
ß*-*actin	*bact*	NM_205518	TGATATTGCTGCGCTCGTTG
			ATACCAACCATCACACCCTGA

The qRT-PCR reactions were performed in a 72-well Rotor-Gene Q using the Rotor-Gene SYBR Green PCR kit according to the manufacturers protocol. Samples were randomly chosen between different time points and treatments for each run to avoid run effects. The C_t_ value and amplification efficiency for each sample was obtained with the Comparative Quantitation Analysis from the Rotor-Gene Q software Four different putative reference genes, Tuba1C, ELF1, 28S, and *ß-actin* were tested for their suitability using the BestKeeper software tool [41]. As *ß-actin* (Table 1) gave the most stable results, it was chosen for reference gene and was included in every run. The amplification efficiency for each gene was determined by taking the average of the amplification efficiency of all samples. The expression of the genes relative to *ß-actin* was calculated according to the Pfaffl method [42].

### Melatonin, GH, and corticosterone analysis

**Fig 2:**
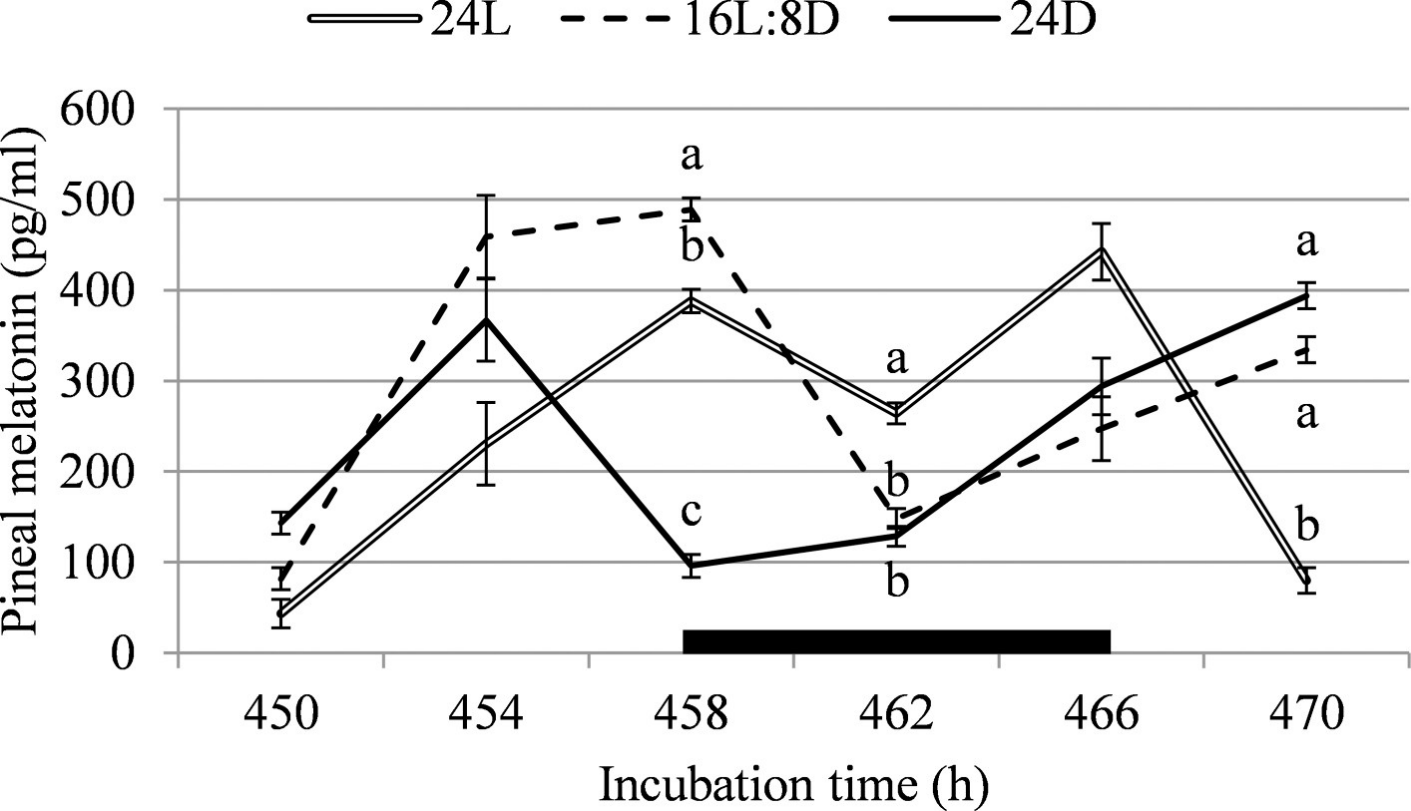
Pineal melatonin between 450 and 470h of incubation, corresponding with light or dark periods for the 16L:8D treatment, in broiler chicken embryos incubated under continuous light (24L), 16h of light, followed by 8h of darkness (16L:8D), or continuous darkness (24D). The black box indicates 16L:8D’s dark period. ^a,b^ Values within a time point with different superscripts differ significantly at *P*≤0.05.

On 450, 454, 458, 462, 466, and 470h of incubation (ED18.75 till ED19.5), 10 embryos per treatment, per time point were sacrificed for blood and pineal gland sampling (N = 180). Time points were chosen to reflect the lighting cycle; 458h was at the start of 16L8D’s dark period, 462h was in the middle of the dark period, 466h was at the start of the light period, 470h was 4 h into the light period of 16L:8D, 450h was 8 h into the light period, and 454h was 12 h into the light period (Fig 2). The egg was opened with rounded tweezers at the blunt end and the egg’s cap was removed to allow easy access to the embryo. Blood was collected directly from the jugular vein of the embryo using a 1-mL heparinized syringe and 30-gauge needle. The blood samples were then transferred to heparin coated tubes on ice. After centrifugation, plasma was stored at -20°C for analysis of GH and corticosterone. Corticosterone was analysed with a double antibody corticosterone 125 RIA kit for rats and mice (ImmuChem Corticosterone DA, Catalogue no. 07–120102; MP Biomedicals, LCC, New York, USA). To be able to use the kit for our chicken samples, it was determined in a preliminary run that the sample had to be diluted from 200x to 5x. GH was analysed with a chicken GH ELISA kit (CSB-E09866Ch; Cusabio, Maryland, USA) following the kit’s instructions. After blood collection, the embryos were decapitated, and pineal glands were removed from the brain using a scalpel and micro dissecting forceps. This was done as quickly as possible to limit the exposure of the embryo to light. The pineal glands were submerged in a 2.0 mL Eppendorf in liquid nitrogen before being stored at -80°C. Pineal melatonin was determined by radio-immunoassay, extracting the pineal glands with absolute methanol prior to the essay, according to Zeman *et al*. [22]. The detection limit of the assay was 1.2 pg/tube and intra- and interassay coefficients of variation for a pooled night time plasma were 7.2 and 9.9, respectively.

### Hatch data

From ED19.75 (474 h of incubation) onward, eggs were checked every 4h, and all hatched chickens were feather sexed, weighed, and numbered individually with a neck label. Their moment of hatch was written down for calculation of total incubation time. Starting at the 12^th^ hatched chicken, every 5^th^ hatched chicken per treatment was sampled for its leg bones until 29 chickens were sampled per treatment (N = 87) to ensure an even spread over the middle of the hatch window. Tibia and femur weight, length, depth, and width were determined in these 87 chickens. The left tibia of every odd numbered chicken was sampled for analysis of gene expression as described previously (n = 6 per treatment). At 510h incubation time, all unhatched eggs were counted to determine hatchability (as a percentage of fertile eggs), and the experiment was terminated.

### Statistical analysis

The overall model used for all data was:

$$Y_{i}=\mu +{Lighting\;schedule}_{i}+\varepsilon i,$$ (1)

where Y_i_ = the dependent variable, *μ* is the overall mean, Lighting schedule_i_ = Incubation lighting schedule (i = 24L, 16L:8D, or 24D), and ε_i_ = the residual error term.

For the analysis of heart rate, 16L:8D data was split up to measurements in its light period (16L:8D light) and in its dark period (16L:8D dark). Incubation time (ED12, ED12.5, ED13 etc. up to ED19.5) was added as a covariable. For the analysis of gene expression, the model was extended with Day (ED13, ED17, or hatch), and its interaction with Lighting schedule. Heart rate, pineal melatonin, plasma corticosterone, and plasma GH data were analysed both per day or time point and overall (averaged over day or time point). All data were analysed with the Glimmix procedure (for fitting generalized linear mixed models) in SAS (SAS Institute, Cary, NC, USA), and the correct distribution type was determined by examining plots of the residuals. Exceptions were hatchability and percentage females, which were analysed using the Logistics procedure for logistic regression analysis of binary response data. The individual embryo or chicken was considered to be the experimental unit. Data are presented as least square means (LSMeans) ±SEM. Differences between treatments were considered to be significant at *P ≤* 0.05.

## Results

### Heart rate and hatch data

Heart rate between ED12 and ED19 was higher for 16L:8D in the light period (283 beats/minute), 16L:8D in the dark period (279 beats/minute), and 24D (279 beats/minute) than for 24L (274 beats/minute) (SEM = 1.6; F = 107.4; *P* < 0.001). Total incubation time till hatch (hatch time) was shorter for 16L:8D than for 24L (-0.6h) and 24D (-2.4h; F = 8.9; *P* < 0.001; Table 2). Body weight at the end of incubation was higher for 24D than for 24L (+1.3%) and 16L:8D (+1.1%; F = 9.4; *P* < 0.001). Hatch of fertile eggs did not differ between treatments (*P* = 0.15). The hatchling sex ratio, tested as percentage of females, did not differ between treatments (*P* = 0.50).

**Table 2:** Hatch time, body weight at hatch, hatch of fertile, and percentage females (as a measure of sex distribution) for broiler eggs and chickens incubated under continuous light (24L), 16h of light, followed by 8h of darkness (16L:8D), or continuous darkness (24D).

	n^1^	Hatch time (h)	n^2^	Body weight (g)	Hatch of fertile (%)	Females (%)
24L	235	497.9^a^	198	46.5^b^	85.7	46.5
16L:8D	235	496.1^b^	212	46.6^b^	89.8	51.9
24D	235	498.5^a^	217	47.1^a^	91.2	47.5
SEM		0.41		0.10		
*P-*value		<0.001		<0.001	0.15	0.50

^1^ Egg was the experimental unit.

^2^ Chicken was the experimental unit.

^a,b^ Values within a column with different superscripts differ significantly at *P*≤0.05.

### Gene expression

No differences were found between 24L, 16L:8D, or 24D in expression of the genes *col1α2*, *col2α1*, *col10α1*, *spp1*, *sparc*, *bglap*, and *alpl*, relative to the reference gene *ß-actin*, in the overall studied period (F < 2.8; P > 0.072) or on any of the measured time points (F < 2.0; P > 0.12; Table 3) or lighting. Irrespective of incubation lighting schedule, *col10α1* expression relative to *ß-actin* was 32.5 to 52.3% higher at hatching than on ED13 and ED17 (F = 7.5; P = 0.002). Relative expression of other genes did not differ between days of incubation (F < 2.5; P > 0.10).

**Table 3:** Gene expression values of collagen type I (*col1α2*), collagen type II (*col2α1*), collagen type X (*col10α1*), osteopontin (*spp1*), osteonectin (*sparc*), osteocalcin (*bglap*), and alkaline phosphatase (*alpl*) relative to *ß-actin* on embryonic day (E)13, ED17, and at hatching for broiler embryos and chickens incubated under continuous light (24L), 16h of light, followed by 8h of darkness (16L:8D), or continuous darkness (24D).

	n^1^	*col1A2*	*col2A1*	*col10a1*	*spp1*	*sparc*	*bglap*	*alpl*
Treatment								
24L	18	1.73	1.61	1.58	1.09	1.25	1.01	1.42
16L:8D	18	1.29	1.16	1.42	1.05	1.32	1.31	1.25
24D	18	1.10	1.08	1.24	1.14	1.07	1.15	1.15
SEM		0.259	0.171	0.190	0.17	0.151	0.142	0.237
Day								
ED13	18	1.62	1.33	0.94^b^	1.40	1.39	1.12	1.43
ED17	18	1.36	1.33	1.33^b^	0.95	1.16	1.34	1.13
Hatching	18	1.14	1.19	1.97^a^	0.92	1.08	1.01	1.25
SEM		0.259	0.171	0.190	0.17	0.151	0.142	0.237
Treatment x day								
24L - ED13	6	2.34	2.10	0.95	1.45	1.82	0.84	2.08
24L - ED17	6	1.78	1.66	1.54	0.99	0.99	1.39	1.15
24L –Hatching	6	1.06	1.07	2.27	0.82	0.93	0.81	1.02
16L:8D - ED13	6	1.34	0.81	0.56	1.44	1.33	1.25	0.98
16L:8D - ED17	6	1.29	1.30	1.41	0.85	1.45	1.60	1.22
16L:8D - Hatching	6	1.25	1.36	2.29	0.85	1.19	1.08	1.57
24D - ED13	6	1.18	1.08	1.32	1.30	1.03	1.27	1.23
24D - ED17	6	1.01	1.03	1.05	1.03	1.05	1.04	1.03
24D –Hatching	6	1.11	1.15	1.35	1.09	1.13	1.13	1.18
SEM		0.449	0.297	0.330	0.294	0.262	0.245	0.410
*P*-values								
Treatment		0.22	0.072	0.45	0.93	0.47	0.34	0.72
Day		0.43	0.81	0.002	0.10	0.33	0.25	0.67
Treatment x Day		0.65	0.12	0.13	0.95	0.26	0.50	0.38

^1^ Embryo was the experimental unit.

^a,b^ Values within a factor, within a column with different superscripts differ significantly at *P*≤0.05.

### Hormones ED18.75 –ED19.5

Pineal melatonin levels differed between incubation lighting schedules on several of the studied time points. At 458h of incubation, which was at the start of 16L:8D’s dark period, pineal melatonin was higher for 16L:8D than for 24L (+20.7%) and 24D (+80.3%), and it was 75.2% higher for 24L than for 24D (F = 63.4; *P* < 0.001; Fig 2). At 462h of incubation, which was in the middle of 16L:8D’s dark period, pineal melatonin was higher for 24L than for 16L:8D (+43.9%) and 24D (+51.3%; F = 10.7; *P* < 0.001). At 470h of incubation, which was 6h into 16L:8D’s light period, pineal melatonin was 76.1% higher for 16L:8D, and 79.7% higher for 24D than for 24L (F = 34.2; *P* < 0.001). Lighting schedule did not affect pineal melatonin content on any other moment (F < 3.4; *P* > 0.05). When data was averaged over all time points, because fluctuations in 24L and 24D were expected to be rhythmic for individuals, but not for the whole group, pineal melatonin did not differ between treatments (F = 1.2; *P* = 0.30).

**Fig 3:**
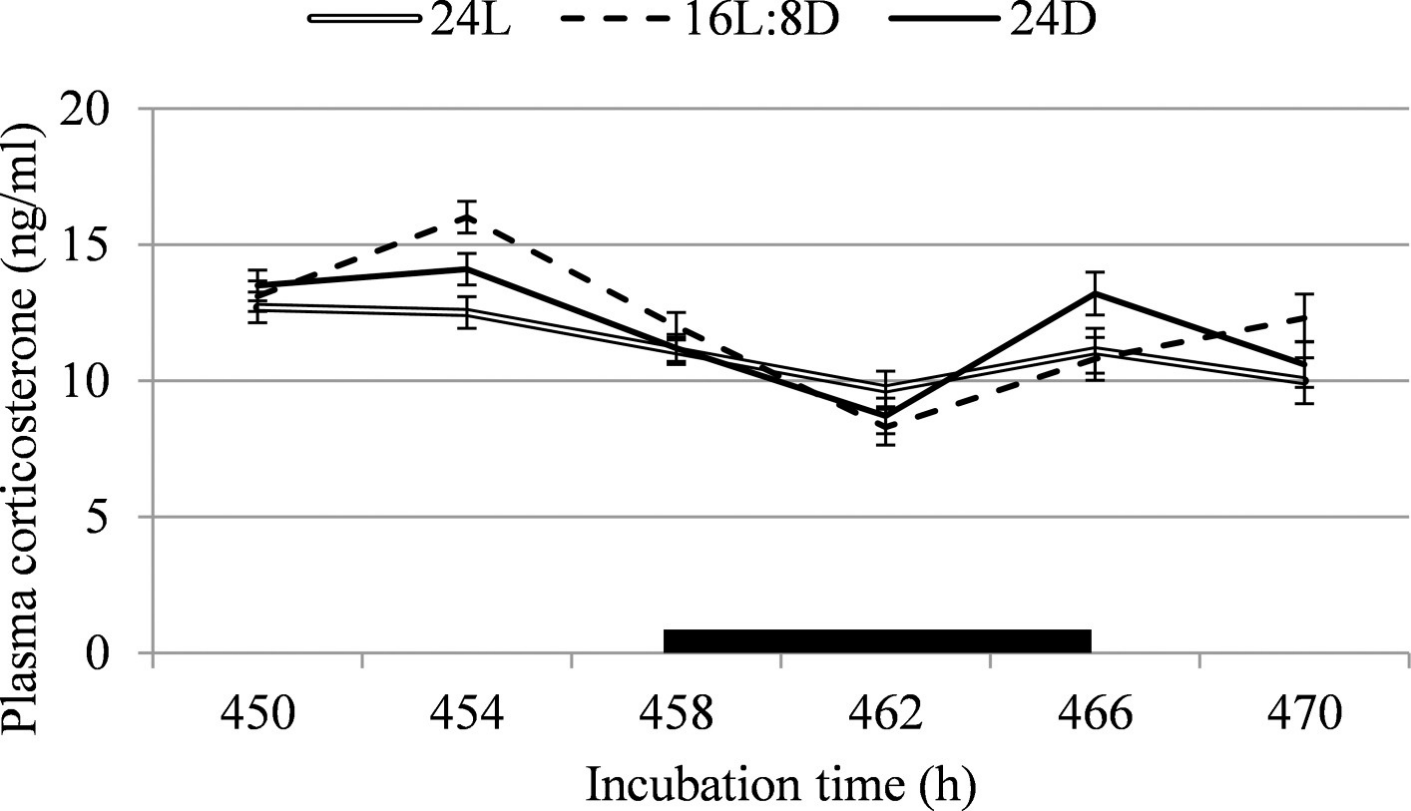
Plasma corticosterone between 450 and 470h of incubation, corresponding with light or dark periods for the 16L:8D treatment, in broiler chicken embryos incubated under continuous light (24L), 16h of light, followed by 8h of darkness (16L:8D), or continuous darkness (24D). The black box indicates 16L:8D’s dark period. ^a,b^ Values within a time point with different superscripts differ significantly at *P*≤0.05.

**Fig 4:**
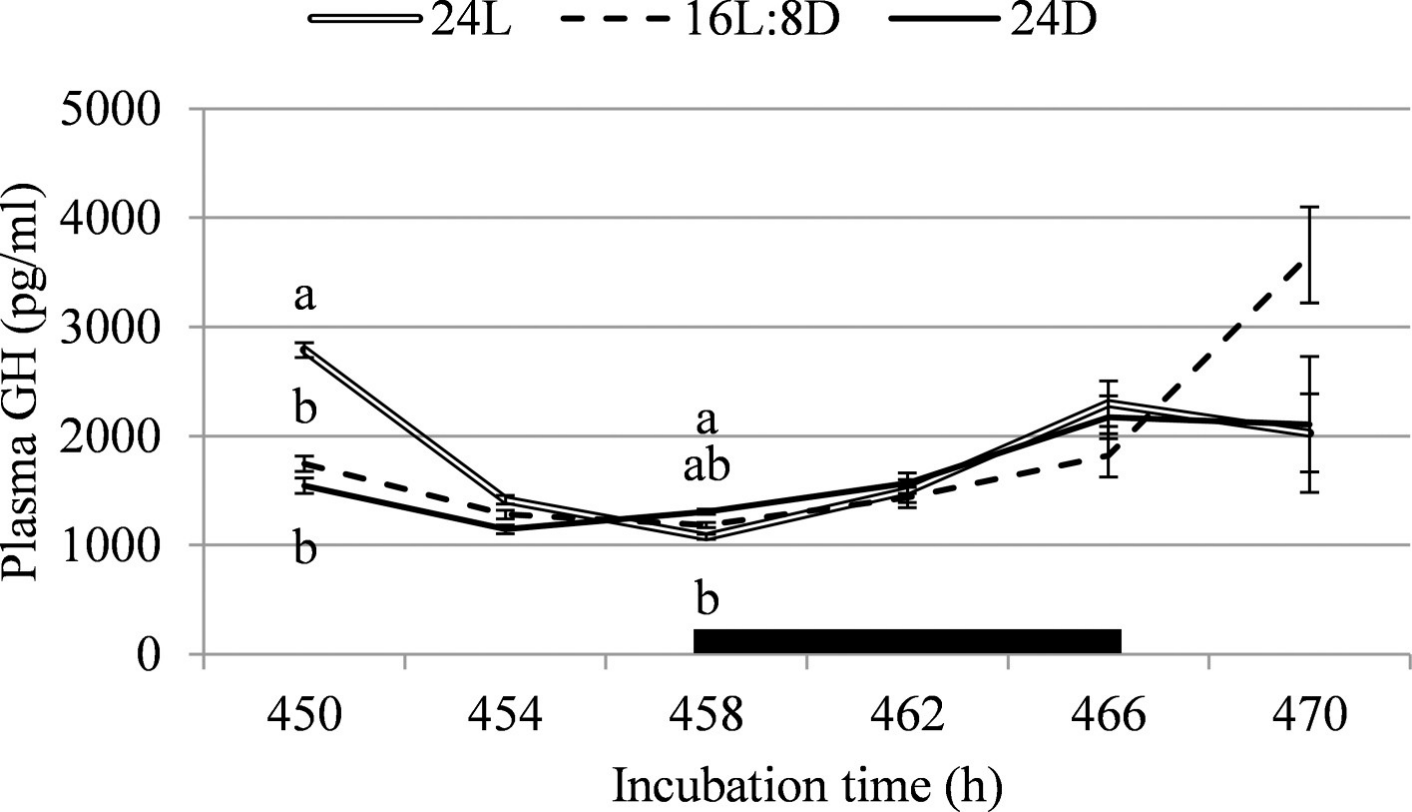
Plasma growth hormone between 450 and 470h of incubation, corresponding with light or dark periods for the 16L:8D treatment, in broiler chicken embryos incubated under continuous light (24L), 16h of light, followed by 8h of darkness (16L:8D), or continuous darkness (24D). The black box indicates 16L:8D’s dark period. ^a,b^ Values within a time point with different superscripts differ significantly at *P*≤0.05.

Corticosterone was not affected by incubation lighting schedule on any sampling point (F < 2.3; *P* > 0.12; Fig 3) or when averaged over sampling points (F = 0.6; *P* = 0.53). At 450h of incubation, 8h into 16L:8D’s light period, GH was higher for 24L than for 16L:8D (+37.4%) and 24D (+44.6%; F = 23.2; *P* < 0.001; Fig 4). At 458h of incubation, at the start of 16L:8D’s dark period, GH was 17.4% higher for 24D than for 24L, with 16L:8D intermediate (F = 6.1; *P* = 0.006). GH was not affected by treatment on any other sampling time (F < 2.9; *P* > 0.073). When data was averaged over all time points, plasma GH did not differ between treatments (F = 1.2; *P* = 0.30).

### Bone dimensions at hatch

Both femur and tibia weight were higher for 24D than for 16L:8D. Femur weight at hatch was 8.0% higher for 24D than for 16L:8D, with 24L intermediate (F = 4.6; *P* = 0.013; Table 4). Tibia weight at hatch was 4.5% higher for 24D than for 16L:8D, with 24L intermediate (F = 3.1; *P* = 0.049). Femur length at hatch was 3.0 to 3.2% higher for 24D than for 16L:8D and 24L (F = 22.0; *P* = 0.013), but tibia length was not affected by incubation lighting schedule (F = 1.1; *P* = 0.35). Both femur and tibia depth resulted in lowest values for 16L:8D. Femur depth was higher for 24L (+3.2%) and 24D (+5.7%) than for 16L:8D (F = 10.3; *P* < 0.001). Tibia depth was higher for 24D than for 24L (+2.3%) and 16L:8D (+6.3%), and it was higher for 24L than for 16L:8D (+4.1%; F = 13.4; *P* < 0.001). Femur width was higher for 24D than for 24L (+3.7%) and 16L:8D (+4.2%; F = 8.8; *P* < 0.001). Tibia width was higher for 24L (+3.8%) and 24D (+5.9%) than for 16L:8D (F = 13.1; *P* < 0.001).

**Table 4:** Weight, length, depth, and width of the femur and tibia at hatch of broiler chickens incubated under continuous light (24L), 16h of light, followed by 8h of darkness (16L:8D), or continuous darkness (24D).

		Weight (g)		Length (mm)		Depth (mm)		Width (mm)	
Treatment	n^1^	Femur	Tibia	Femur	Tibia	Femur	Tibia	Femur	Tibia
24L	29	0.217^a^^b^	0.331^a^^b^	21.94^b^	30.93	1.87^a^	1.71^b^	1.82^b^	1.82^a^
16L:8D	29	0.207^b^	0.321^b^	21.99^b^	31.00	1.81^b^	1.64^c^	1.81^b^	1.75^b^
24D	29	0.225^a^	0.336^a^	22.67^a^	31.17	1.92^a^	1.75^a^	1.89^a^	1.86^a^
SEM		0.0044	0.0043	0.089	0.120	0.017	0.016	0.015	0.016
*P*-value		0.013	0.049	<0.001	0.35	<0.001	<0.001	<0.001	<0.001

^1^ Chicken was the experimental unit.

^a,b,c^ Values within a column with different superscripts differ significantly at *P*≤0.05.

## Discussion

At hatch, femur and tibia weight, depth, and width, and femur length were higher for 24D than for 16L:8D. 24L also showed smaller bone dimensions than 24D, with lower femur length, tibia depth, and femur width. The same held when body weight was added to the statistical model as a covariable in exploratory analysis to correct for the fact that larger chickens may have larger bones. The reducing effect of 16L:8D on bone dimensions was unexpected, considering our previous research where 12L:12D resulted in a longer femur at hatch than both 24D and 24L, and 24D and 12L:12D resulted in a longer and heavier tibia than 24L [3]. The lighting source in the present experiment was the same as in the previous experiment, and eggs were of the same breed and a similar parent flock age. It can be hypothesized that the length of the dark period should be more than 8 hours, as that seems to be the largest difference between the 16L:8D and 12L:12D treatments. 16L:8D was chosen as a lighting schedule comparable to what a chicken might experience post hatch. However, during incubation, chicken embryos are exposed to fewer hours of light. In nature, hens will not leave the nest daily, and when they do, they are off the nest for 15 minutes to 1.5 hours [43]. Combined with the fact that 24L was found to result in reduced bone dimensions at hatch compared to 24D, it can be speculated that exposure to too many hours of light (in this case, 16 or 24 hours per day) during incubation is detrimental for leg bone development, although a lighting schedule with longer dark periods may be beneficial [3].

Increased bone dimensions do not necessarily signify increased bone strength. It can be speculated that larger bones signify stronger bones. We previously found lower tibial cortical area, second moment of area, and mean cortical thickness for 24L than for 12L:12D and 24D [3]. According to Augat and Schorlemmer [4], these geometrical measures are up to 70–80% predictive of whole bone strength. The present study found higher tibia and femur width for 24D than for 16L:8D, and higher femur width at hatching for 24D than for 24L. Cortical width is another strong predictor of bone strength [4]. Unfortunately, we did not measure bone breaking strength in the present experiment, so it is not known whether these increases in bone width indeed resulted in higher bone strength, and whether this continued to be so post hatching.

In our previous study, we found a longer ossified portion of the tibia for broiler embryos incubated under 12L:12D than for embryos incubated under 24L on ED13 and ED14 [3]. We therefore expected to find higher expression of genes related to cartilage (*col1α2*, *col2α1*, *col10α1*) and bone development (*spp1*, *sparc*, *bglap*, and *alpl)* for tibias of broiler embryos incubated under 16L:8D. However, we did not see any differences between incubation lighting treatments in expression of these genes on ED13, ED17, or at hatch. It is possible that 24D (which lead to the highest bone dimensions at hatching) showed higher embryonic ossification, but this was not measured in the present study.

There are several explanations for the lack of an effect of incubation lighting schedules on bone gene expression, despite the effect on morphology at hatch. Although the involvement of the studied genes in bone development and maturation has been established before [7–19], it is possible that the stimulating effect of 24D compared to 24L and 16l:8D did not take place through upregulation of these specific genes. We looked at collagenous proteins and proteins related to chondrocytes and osteoblasts as we hypothesized that these may be involved in the effects found on embryonic bone development. However, Rath et al. [44] emphasize the complexity of the maturation of bone, as many molecular and biomechanical changes are involved. Other proteins known to be involved in bone formation include Indian hedgehog [45], RUNX2 [46], and bone morphogenic proteins [47]. It could be interesting to include more genes related to bone development in a follow-up study.

Another possibility is that the wrong bone region was sampled. Whole tibias were sampled in the present experiment, but it is possible that expression of the genes measured in this study was localized, and sampling the whole bone did not reveal localized upregulation. The epiphyseal plate seems to be a relevant region, as osteopontin [11,14], osteocalcin [14], osteonectin [17], and alkaline phosphatase [11,19] are all found in the hypertrophic or mineralizing zones of the epiphyseal plate. Possibly, sampling only the primary ossification centres, or only the epiphyseal plates, may reveal differences in gene expression.

A third possibility is that the upregulating effect of lighting conditions during incubation took place before we started sampling the tibiae. First ossification of chicken leg bones occurs from approximately ED9 onward in primary ossification centres in the middle of the bone’s diaphysis [9]. In the primary ossification centres, cartilage is replaced by bone marrow, while collagen type 1 rich osteoid forms a bone collar around the cartilage model. The osteoid later becomes mineralized [7,8]. Sampling of bones for ossification measurements stopped at ED14 in the previous study [3], and sampling for gene expression started only by ED13 in the current study. It is known that ossification is incomplete at hatch [9], but it is possible that any effects of incubation lighting conditions on bone development took place prior to ED13.

A pathway that can affect bone development at hatch is embryonic activity, as movement stimulates bone development in chicken embryos [32,33]. Heart rate readings were used as a non-invasive measurement of activity in the present experiment. In human foetuses, higher activity is positively related with heart rate [48]. In chicken embryos, Noiva *et al*. [49] found higher heart rate for embryos when they were inactive than for moving embryos. However, this was likely a response to overheating. When incubated under high air temperatures (38.9°C), Noiva *et al*. [49] found increased heart rate, but decreased movements in broiler embryos compared to incubation at an air temperature of 37.8°C. In the present experiment, overheating of the embryos was prevented by incubating the eggs at an eggshell temperature, as a reliable reflection of embryo temperature [50], of 37.8°C throughout incubation. Heart rate in the present experiment was lower for 24L than for 16L:8D, both in its dark and light period, and 24D. This would suggest that 24L incubated broiler embryos were less active than 16L:8D and 24D incubated embryos. Moriya *et al*. [51] incubated eggs in an incubator with glass doors and allowed natural light to enter the incubator, resulting in a circadian light-dark rhythm. They did not find a clear effect of light or dark period, either [51]. Because leg bone development was advanced for 24D compared to 16L:8D, it seems that the relation between heart rate as a reflection of embryonic activity and bone development at hatch is not completely clear from the present experiment.

In literature, a clear pattern in pineal melatonin concentration for chicken embryos exposed to a lighting schedule during incubation is described, with peaks in the dark period [22,52]. The differences in pineal melatonin concentrations found in the present experiment did not follow a logical pattern over 16L:8D’s light and dark periods, or overall between treatments. Pineal melatonin in 16L:8D appears to increase 4 hours before the start of the dark period, which could have been suggestive of synthesis and storage of pineal melatonin, which was later released into the plasma when the dark period started. However, literature does not support this, as pineal melatonin was not found to start increasing already 4 hours before the onset of darkness [53], and concentration of plasma melatonin closely follows pineal melatonin’s rhythm [22]. Possibly, we did not find a rhythm in pineal melatonin because embryos were exposed to light for too long during blood sampling before pineal extraction; it is not known how quickly melatonin levels degraded in the chicken pineal gland *in vivo* after light exposure. Plasma GH was expected to follow a rhythm closely resembling that of pineal melatonin, as GH has been shown to peak along with melatonin release [24,54]. However, results from the present study showed that the plasma GH pattern was very different from the pattern of pineal melatonin concentration. Previous studies did find a clear stimulating effect of light during incubation on plasma GH levels, but these studies were mostly performed with green light [26,27] whereas white light was used in the present study. Corticosterone, which was measured a as a possible indicator of stress, did not differ between treatments. Overall, the endocrine pathway did not provide a clear explanation for the differences between incubation lighting treatments in leg bone development that we found at hatch.

Upregulation of gene expression, increased activity as measured through embryonic heart rate, and endocrine levels were all not found to show a clear pathway through which bone development was increased at hatching for 24D compared to 24L and 16L:8D, especially. Other pathways that were outside of the scope of this study can be speculated to have been involved. For example, post hatching, the importance of nutrition on bone development has been well established [55]. In our previous study, we found a higher liver and intestines weight at hatching for 12L:12D than for 24L [3], and [56] found higher residual yolk weight at hatching for embryos incubated under 24D than 24L of monochromatic green light. If lighting conditions during incubation affect the development of digestive organs and yolk uptake, it is possible that they also have an effect on nutrient uptake and utilization, which may indirectly impact bone development. Unfortunately, this was not studied in the current setup.

The same setup as described in the current experiment was additionally used to incubate eggs that were grown to 35 days of age after hatching in another experiment [36]. At day 35, the incidence of epiphyseal plate abnormalities was found to be increased for 24L compared to 16L:8D and 24D, and the incidence bacterial chondronecrosis with osteomyelitis was found to be increased for 24D compared to 16L:8D [36]. This suggests that the increased bone dimensions found for 24D at hatching in the present experiment do not necessarily lead to improved leg bone health at slaughter age, and cannot be used as a predictor of leg health.

## Conclusions

To conclude, applying 16 or 24 hours of white LED light per day during incubation appeared to reduce tibia and femur dimensions at hatch compared to dark incubation in broiler chickens, suggesting that more than 8 hours of darkness per day may be necessary for highest embryonic bone development. Several pathways that may have explained this difference in bone development were investigated, but based on gene expression the involvement of collagenous proteins and proteins related to chondrocytes and osteoblasts, melatonin, growth hormone, corticosterone, and embryonic activity as measured through heart rate was not proven.

## Supporting information

S1 Dataset Data for gene expression (“Genes” tab), embryonic heart rate (“HeartRate” tab), embryonic pineal melatonin (“Melatonin” tab), embryonic plasma growth hormone (“GH” tab) and corticosterone (“CORT” tab), hatch of fertile eggs (“HatchOfFertile” tab), chick data at hatching (“HatchData” tab), and leg bone dimensions at hatching (“Bones” tab). (XLSX)
